# The Utility and Appropriateness of Single-Position Circumferential Lumbar Interbody Fusion Using O-Arm-Based Navigation in the Novel Oblique Position

**DOI:** 10.3390/jcm12227114

**Published:** 2023-11-15

**Authors:** Tetsuro Ohba, Kotaro Oda, Nobuki Tanaka, Hirotaka Haro

**Affiliations:** Department of Orthopaedic Surgery, University of Yamanashi, 1110, Shimokato, Chuo, Yamanashi 409-3898, Japan; koda@yamanashi.ac.jp (K.O.); tanakan@yamanashi.ac.jp (N.T.); haro@yamanashi.ac.jp (H.H.)

**Keywords:** minimally invasive technique, oblique position, percutaneous pedicle screw, O-arm, CT navigation, accuracy, lateral interbody fusion

## Abstract

Purpose: Single-position surgery with patients in a lateral position, which involves inserting percutaneous pedicular screws (PPS) and lateral interbody fusion (LIF) to avoid changing the position, has been reported. The purpose of the present study was to evaluate the utility and appropriateness of single-position LIF-PPS using O-arm-based navigation in the innovative oblique position. Methods: This study involved a retrospective analysis of 92 consecutive patients with lumbar spondylolisthesis who underwent LIF-PPS using O-arm-based navigation. Thirty-five subjects demonstrated surgery with repositioning, as well as 24 in the lateral decubitus position, and 33 in the oblique during PPS, where the position was changed to the lateral decubitus position using bed rotation without resetting. We compared these three groups in terms of the surgery time, blood loss, and the accuracy of the screw placement. Results: The operative time was significantly shorter in the single-position surgery, both in the lateral and oblique positions, compared to surgery in a dual position. The blood loss was significantly increased in the lateral position compared to the dual and oblique positions. The screw trajectory angle on the downside was significantly smaller in the lateral position, and the accuracy of the screw placement on the downside was significantly lower in the lateral position compared to the dual and oblique positions. Conclusion: Single-position surgery could reduce the average surgery time by about 60 min. The present study indicated the oblique position during PPS insertion might make single-position surgery more useful to improve the accuracy of PPS on the downside, with less blood loss.

## 1. Introduction

Spinal fusion is a popular surgical technique for stabilizing the intervertebral space to treat patients with spinal instability, deformity, degenerative scoliosis, or lumbar canal stenosis. Traditional open posterior approaches for lumbar fusion might require extensive dissection of the paraspinal muscles, which can result in permanent muscle denervation, loss of function, late onset of spinal instability, and low back pain [1]. Open lumbar spine surgeries have been reported as a possible cause of surgical site pain compared to minimally invasive techniques [2]. Lateral interbody fusion with posterior percutaneous pedicle screw (LIF-PPS) fixation has been widely performed as a more modern and less invasive approach for the lumbar interbody fusion technique. Advantages of LIF-PPS compared to the traditional open procedure have been reported, including strong correction, a strong fixation force, significantly less bleeding, less invasion of the back muscles, and fewer neurological and operative complications [3,4,5,6]. Conventionally, this procedure was performed LIF, with the patient in a dual lateral decubitus, and PPS with a prone position [7]. Changing positions under anesthesia in the middle of a surgical procedure can prolong the time in the operating room and risk problems with the ventilator and various medical codes. Single-position surgery with patients in a lateral position comprises inserting PPS and performing LIF to avoid a changing position [8,9,10,11]. A recent systematic review revealed that the literature comparing a single-position versus lateral-then-prone lumbar fusion showed a shorter time spent in the operating room and in hospital stays while maintaining comparative rates of perioperative outcomes, complications, and correction of local lordosis [12,13]. Nevertheless, there are remaining concerns about increased interoperative blood loss during PPS and the risk of screw misplacement due to lateral-position surgery [9,14]. Therefore, using bed rotation, we modified a single-position LIF-PPS procedure with O-arm-based navigation to a near-supine position for PPS insertion (named oblique position) and then to a lateral position for LIF.

To assess the utility and appropriateness of the single-position LIF-PPS using O-arm-based navigation in the innovative oblique position, we conducted a retrospective comparative study of the LIF-PPS procedure performed with the patients in dual, lateral decubitus, and oblique positions.

## 2. Methods

### 2.1. Patient Group

The study was approved by the Ethics Committee of our university and was performed with written informed consent from all eligible patients. Patients were candidates for LIF-PPS surgery if lumbar fusion was needed because of degenerative lumbar spondylolisthesis, and if a full course of conservative care, such as therapeutic exercise, drug, and brace treatments, had been exhausted. The following exclusion criteria were applied: (1) fusion length >3 intervertebral segments; (2) history of past lumbar surgery; and (3) degenerative scoliosis (coronal curve >30°). We retrospectively analyzed data from 92 consecutive patients with lumbar spondylolisthesis who underwent LIF-PPS using O-arm-based navigation from three board-certified spinal surgeons at our institute from 2018 to 2022. The dual-position (DP) group included data from 35 patients and 198 screws, the single lateral decubitus position (LD) group included data from 24 patients and 148 screws, and the single oblique (O) position during PPS included data from 33 patients and 188 screws. Operative times and blood loss were noted from the medical records. The performance status (PS) established by the Eastern Cooperative Oncology Group was recorded by the attending physician from the day after surgery to 7 days after surgery.

### 2.2. Surgical Technique for the Dual-Position (DP) Group

The patients were placed in a lateral decubitus position on a Jackson table (Mizuho OSI, Union City, CA, USA) using a trunk indicator and medical tape for trunk fixation during the LIF procedure. After inserting two pins into the iliac crest to fix the reference frame, an O-arm scanner (Medtronic, Louisville, CO, USA) was then operated to obtain intraoperative CT images of the lumbar spine, and the data were transferred to a computer navigation system (Stealth Medtronic Navigation, Louisville, CO, USA). Navigation tools were registered. In all cases, artificial bone was used for the grafted bone in the LIF cage, and resection of iliac bones for bone graft was not performed. After the LIF cage was inserted and the wound was closed, the patients were repositioned prone on a Jackson table in the standard manner, and surgical drapes and sterile instrument fields were reset. After fixing the reference frame by clamping the spinous process with a clamp, the O-arm scanner was used again after repositioning. Posterior PPS fixation using intraoperative CT image-guidance navigation was performed as previously described [15].

### 2.3. Surgical Technique for the Lateral Decubitus Position (LD) Group

The patients were placed in a lateral decubitus position on a Jackson table using a trunk indicator and medical tape for trunk fixation, in the same way as the DP group. Fixation of the reference frame was achieved by clamping the spinous process at one or two spinal levels superior to the fusion level. First, two surgeons performed posterior PPS fixation from the ventral and dorsal sides to avoid navigation errors caused by intervertebral expansion and/or correction associated with the cage insertion of the LIF. Following PPS insertion, the LIF procedure was performed from the patient’s ventral side using intraoperative CT image-guidance navigation in the same position without an additional O-arm scan. A reset of surgical drapes and sterile instrument fields were not needed.

### 2.4. Surgical Technique for the Oblique (O)-Position Group

**Figure 1:**
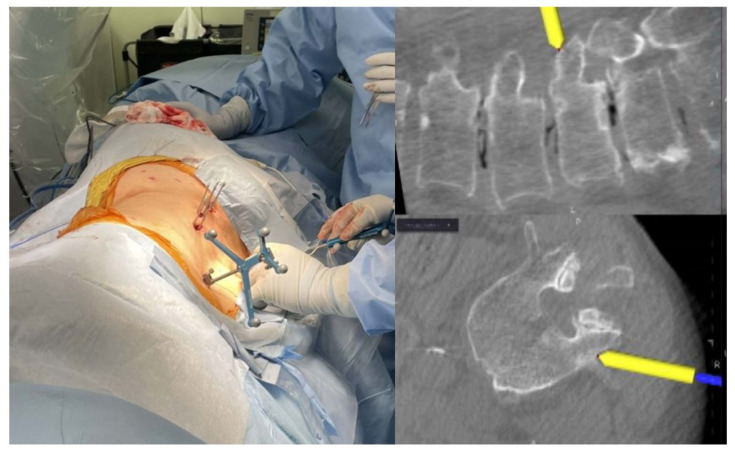
Photographs and the intraoperative CT navigation screen during PPS insertion in the oblique group, where the patients were placed in an oblique position of approximately 45° on a Jackson table. PPS, percutaneous pedicle screw. Yellow arrow denote Navigation pointer.

The patients were placed in an oblique position of approximately 45° on a Jackson table, fixing the trunk more firmly compared to the lateral decubitus position with medical tape to prevent the trunk from shifting position during the bed rotation (Figure 1). Fixation of the reference frame was achieved by clamping the spinous process at one or two spinal levels superior to the fusion level. First, two surgeons performed posterior PPS fixation from the ventral and dorsal sides to avoid navigation errors caused by intervertebral expansion and/or correction associated with the cage insertion of the LIF. Following PPS fixation in an oblique position, the patient was repositioned to a lateral decubitus position using bed rotation without resetting surgical drapes and sterile instrument fields. Then, the LLIF procedure was performed from the patient’s ventral side using intraoperative CT image-guidance navigation in a lateral decubitus position.

### 2.5. Radiological Evaluation

All patients underwent postoperative imaging using an eight-slice multidetector CT system with a 0.83 mm section thickness (Lightspeed Ultra; GE Healthcare, Milwaukee, WI, USA). The trajectory angle of the pedicle screws at each lumbar vertebral level was measured in axial CT images, as previously described [16]. All evaluations of accuracy were performed by two independent spinal surgeons who were blinded to the conditions. Screw positioning was evaluated via axial, sagittal, and coronal plane images to determine whether screw breaches occurred medially, laterally, inferiorly, or superiorly. The accuracy of the screw placement was evaluated according to criteria established previously [17].

### 2.6. Statistical Analysis

Continuous variables were compared using unpaired *t*-tests, and categorical variables were assessed using Fisher’s exact tests for baseline characteristics among groups. Mean ± SD values were denoted for continuous variables, or number (percentage) values were used for categorical variables. We conducted Student’s *t*-test, or Kruskal–Wallis (to compare three groups), Mann–Whitney, or Fisher exact tests to compare the mean values between the groups (dual position, lateral position, and oblique position), assuming normal distributions for continuous variables. We used Prism (version 9.0; GraphPad Software, La Jolla, CA, USA) to calculate the summary statistics and conduct the *t*-tests. Asterisks indicate statistical significance (*p* < 0.05).

## 3. Results

The preoperative baseline characteristics of the patients who underwent minimally invasive surgery spinal fusion with LIF-PPS using O-arm-based navigation are summarized in Table 1. There was no significant difference in the patient mean age (DP group; 70.2 ± 9.3 LD group; 72.7 ± 12.0 O group; 73.0 ± 11.8, *p* = 0.26), sex, average body mass index (BMI), or fusion length among groups. The average fusion length in each group was 2.06 ± 0.69 (DP group), 1.75 ± 0.85 (LD group), and 1.83 ± 0.89 (O group).

**Figure 2:**
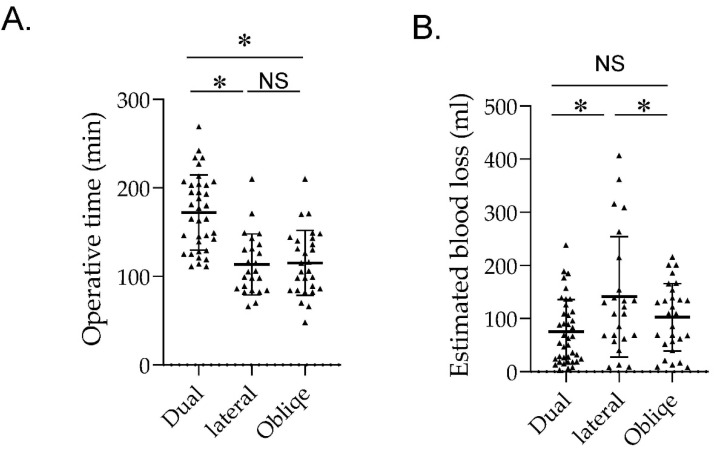
(**A**) Graph comparing the operative time among groups. (**B**) Graph comparing the estimated blood loss among groups. * *p* < 0.05. NS; not significant.

The operative time was significantly longer for patients in the DP group than for patients in the LD and O groups (172.1 ± 42.5 min vs. 113.5 ± 34.4 min vs. 1125.5 ± 36.7 min, respectively; *p* < 0.001). The estimated blood loss was significantly increased in the LD group compared with the DP and O groups (140.9 ± 113.3 mL vs. 75.1 ± 60.7 mL vs. 102.6 ± 113.3 mL, respectively; *p* < 0.001). There was no significant difference in the estimated blood loss between the DP and O groups (Figure 2 and Table 2). The postoperative recovery of PS was not significantly different between the three groups. There were no cases in the single-position group that required an O-arm re-imaging.

The radiographic outcomes (screw trajectory angles and accuracy of PPS) of patients in the three groups are shown in Table 2. There was no significant difference in the screw trajectory between groups on the upside (34.8 ± 7.1° vs. 34.2 ± 7.6° vs. 34.1 ± 5.6°, respectively; *p* = 0.9). In contrast, the screw trajectory on the downside was significantly smaller in patients in the LD group than in patients in the DP and O groups (30.1 ± 6.1° vs. 33.9 ± 6.6° vs. 33.6 ± 6.2°, respectively; *p* < 0.05). Overall, the frequency of no misplaced screws (Grade 1) in patients in the LD group was significantly lower than that in patients in the DP and O groups (125/148 (84.4%) vs. 181/198 (91.4%) vs. 173/188 (92%), respectively; *p* < 0.05). For the upside screws, there was no significant difference in the accuracy between the groups. In contrast, the frequency of no misplaced downside screws was significantly lower in patients in the LD group than in patients in the DP and O groups (58/74 (78.4%) vs. 90/94 (91.0%) vs. 87/94 (92.6%), respectively; *p* < 0.05). Grade 3 misplaced screws were only seen upside in patients in the LD group (4/94, 4.3%). There were no occurrences of neurovascular injury due to screw placement in either group, and no revision surgery was needed in these groups.

## 4. Discussion

**Figure 3:**
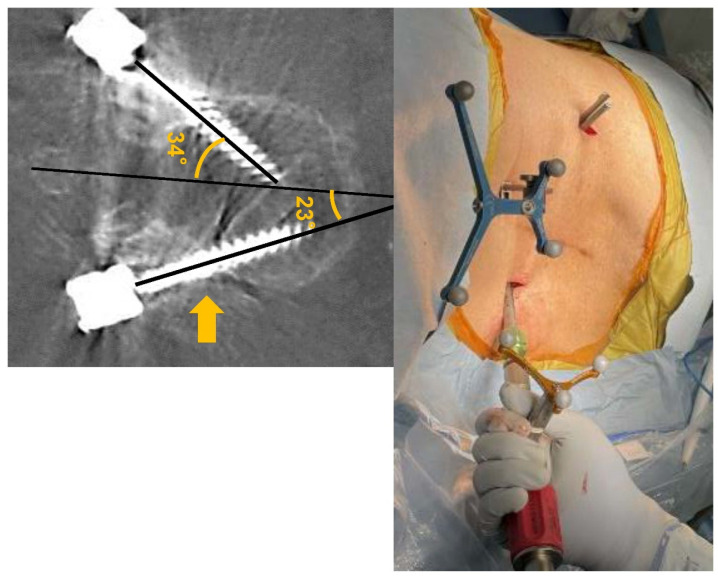
Axial CT and photographs of a representative case of the PPS downside placement in the lateral position; attention was needed to avoid a lateral breach. PPS, percutaneous pedicle screw. Yellow arrows indicate deviation outside of screw.

Knowledge of the effectiveness and safety of LLIF and PPS using intraoperative CT image-guidance navigation has been reported in recent years and is rapidly spreading [9,18,19]. Nevertheless, concerns about increasing the exposure of patients to radiation by using CT image-guided navigation instead of traditional guidance with fluoroscopy (C-arm) have been reported [20]. Conventional LIF-PPS fixation with CT image-guided navigation with patients in two (dual) positions requires at least two scans. In the present study, there were no patients in the single-position group who required reimaging with an O-arm, and all patients underwent surgery after a single scan. This result indicated that single-position surgery also had advantages in terms of reducing patient exposure. Consistent with previous reports, a single-position procedure reduces surgical time by approximately 60 min compared with the conventional dual-position procedure. Additionally, eliminating the need for intraoperative repositioning to the prone position is associated with reduced medical costs and complications such as postoperative vision loss and cardiovascular complications, including hypovolemia and cardiac arrest [21,22]. Because of these advantages, the single-position procedure is currently attracting attention as a useful method. However, concerns remain about the pitfalls and the surgeon’s learning curve for PPS placement in the lateral position [23]. Recent reports have indicated that for PPS downside placement in the lateral position, attention was needed to avoid a lateral breach, which was considered to be related to the inability of the surgeon to place the screws medially due to obstruction from the operative bed [24,25]. Our data also showed a significantly lower accuracy for PPS downside placement in the LD group (Figure 3). Another concern for lateral position surgery was increased blood loss, because gravity makes it easier for blood to drip [9]. An advantage of intraoperative CT navigation is that there is no need to adjust the fluoroscopic image after bed rotation. Recently, single-position surgery for lumbar circumferential fusion with patients in various positions has been suggested, such as prone-position LLIF, lateral-position ALIF, and their combination [26]. We considered that bed rotation could be useful to achieve a more advantageous surgical position for each procedure that PPS demonstrated in the oblique position and LIF demonstrated in the lateral position, and the present study indicated an oblique position during PPS insertion that might make single-position surgery more useful to improve the accuracy of the PPS downside placement and to reduce blood loss. Numerous studies have compared single-position surgery and conventional surgery with repositioning for LIF-PPS for lumbar fusion. But among them, to our knowledge, the present study is the first to compare a single lateral position and oblique position during PPS in LIF-PPS for lumbar fusion. Another concern with this procedure was the accuracy of navigation in single lateral or oblique position. Few studies have examined the accuracy and reality of intraoperative CT navigation in lateral approaches to the spine [18]. In our previous study, we reported that most navigation errors in spinal surgery using intraoperative O-arm navigation occur when the position of the reference frame shifts during the surgical procedure near the reference frame [27]. Based on these experiences, we devised a procedure to fix the reference frame strongly to the spinous processes 1–2 vertebrae cephalad of the intervertebral body to be fixed and to perform PPS prior to LIF to avoid the navigation errors caused by intervertebral expansion and/or correction associated with the cage insertion of the LIF. As a result of those efforts, there were no cases in the present study in which the cage or screw insertion position was misaligned due to navigation errors, and there were no cases in which O-arm reimaging was necessary.

The present study had some limitations. First, this was a retrospective study using data based on medical records with a small sample size. Second, the present study did not include clinical data such as the fusion rate, correction of lumbar lordosis, and the postoperative quality of life of the patients. However, the present study is clinically significant, as it shows the utility and appropriateness of the single-position LIF-PPS using O-arm-based navigation in the innovative oblique position, including lower patient radiation exposure, a shorter operation time, and sufficient screw insertion accuracy.

## 5. Conclusions

The operative time was significantly shorter in single-position surgery both in the lateral and oblique position compared to the dual position, but the blood loss was significantly increased and the accuracy of the screw placement on the downside was significantly lower in the lateral position compared to the dual and oblique positions. Single-position circumferential lumbar interbody fusion using O-arm-based navigation in an innovative oblique position during PPS insertion might make single-position surgery more useful to improve the accuracy of the PPS downside placement and to reduce blood loss.

## Figures and Tables

**Table 1:** Preoperative demographics of patients.

		Single Position		*p*
	Dual Position (*n* = 35)	Lateral (*n* = 24)	Oblique (*n* = 33)	
Age * (years)	70.2 ± 9.3	72.7 ± 12.0	73.0 ± 11.8	0.26
Sex (female/male)	24/11	19/5	26/7	0.53
BMI (kg/m^2^)	23.1 ± 3.4	23.3 ± 4.7	22.8 ± 4.1	0.21
Fusion length *	2.06 ± 0.69	1.75 ± 0.85	1.83 ± 0.89	0.19
Fused level of vertebra				
L2–3	8	4	7	
L3–4	9	6	8	
L4–5	34	23	31	

BMI, body mass index. * Mean ± standard deviation (SD).

**Table 2:** Surgical invasion and pedicle screw parameters.

		Single Position		*p*
	DP (*n* = 35)	LD (*n* = 24)	O (*n* = 33)	
Operative time (min)	172.1 ± 42.5 *	113.5 ± 34.4	115.2 ± 36.7	<0.001
Estimated blood loss (mL)	75 ± 60.6	140.9 ± 113	102.6 ± 63.6	<0.05
Screw parameters	*n* = 198	*n* = 148	*n* = 188	
Screw trajectory angle (°)				
Upside	34.8 ± 7.1	34.2 ± 7.6	34.1 ± 5.6	0.9
Downside	33.9 ± 6.6	30.1 ± 6.1 *	33.6 ± 6.2	<0.05
Accuracy of PPS placement (Grade) ^†^				
Grade 0 (Upside)	91 (91.9)	67 (90.5)	86 (95.5)	0.95
Grade 1	6 (6.1)	4 (5.4)	4 (4.3)	0.85
Grade 2	2 (2.0)	3 (4.1)	4 (4.3)	0.66
Grade 3	0	0	0	NS
Grade 0 (Downside)	90 (91.0)	58 (78.4) *	87 (92.6)	<0.05
Grade 1	7 (7.2)	6 (9.7)	4 (4.3)	0.58
Grade 2	2 (2.0)	6 (9.7)	3 (3.2)	0.16
Grade 3	0	4 *	0	<0.05

DP, dual position; LD, lateral decubitus; O, oblique; PPS, percutaneous pedicle screw. Mean ± standard deviation (SD). ^†^ Values are the number of screws (%). * *p* < 0.05. NS; not significant.

## Data Availability

The data presented in this study are available on request from the corresponding author.
